# Erratum: On the impacts of the COVID-19 pandemic on mortality: Lost years or lost days?

**DOI:** 10.3389/fpubh.2023.1172636

**Published:** 2023-03-09

**Authors:** Frontiers Production Office

**Affiliations:** Frontiers Media SA, Lausanne, Switzerland

**Keywords:** all-cause mortality, COVID-19, life expectancy, population life loss, remaining life expectancy, years lost

An omission to the funding section of the original article was made in error. The following sentence has been added: “Open access funding was provided by the University of Lausanne.”

The original article has been updated.

